# Genotyping and sero-virological characterization of hepatitis B virus (HBV) in blood donors, Southern Ethiopia

**DOI:** 10.1371/journal.pone.0193177

**Published:** 2018-02-20

**Authors:** Henock Ambachew, Meijuan Zheng, Faustina Pappoe, Jilong Shen, Yuanhong Xu

**Affiliations:** 1 Department of Clinical Laboratory, First Affiliated Hospital, Anhui Medical University, Hefei, Anhui, China; 2 Department of Clinical Laboratory Diagnostics, First Affiliated Hospital, Anhui Medical University, Hefei, Anhui, China; 3 Department of Medical Laboratory Sciences, College of Medicine and Health Sciences, Hawassa University, Hawassa, Ethiopia; 4 Department of Immunology and Parasitology, Provincial Laboratory of Microbiology and Parasitology and the Key Laboratory of Zoonoses Anhui, Anhui Medical University, Hefei, Anhui, China; 5 Department of Microbiology and Immunology, School of Medical Sciences, College of Health and Allied Sciences, University of Cape Coast, Cape Coast, Ghana; University of Cincinnati College of Medicine, UNITED STATES

## Abstract

Hepatitis B virus (HBV) prevalence is highest in Sub-Saharan Africa including Ethiopia. HBV genotypes have distinct geographic distributions and play a role in course of infection and treatment management. However, in Ethiopia there is paucity of information about distribution of HBV genotypes. This study was done to determine genotype, mutation and sero-virological profiles of HBV isolates in Southern Ethiopia. Cross-sectional, laboratory based study was conducted on 103HBsAg sero-positive samples from a total of 2,237 screened blood donors. HBV serological markers and biochemical assays were done. Serum viral load was measured using quantitative real-time PCR. Partial HBV S-gene was amplified with nested PCR and sequenced. Bioinformatics tools were utilized to determine genotypes, serotypes and mutations. Of 103 HBsAg reactive serum samples, 14.6% and 70.9% were sero-positive for HBeAg and HBeAb, respectively. Ninety-eight samples gave detectable viral load with a median of 3.46(2.62–4.82) log IU/ml. HBeAg sero-positive donors carried elevated levels of viral load. Eighty five isolates were successfully amplified, sequenced and genotyped into 58 (68.2%) genotype A (HBV/A) and 27 (31.8%) genotype D (HBV/D). HBV serotypes found were *adw2* (74.1%), *ayw2* (24.7%), and *ayw3* (1.2%). In twenty-four (28.2%) samples mutations in the major hydrophilic region (MHR) were observed. Donors infected with HBV/A had higher viral load and more frequent MHR mutation than HBV/D infected donors. This study illustrated distribution of HBV genotype A and D among blood donors in southern Ethiopia. It also demonstrated occurrence HBV variants that may influence clinical aspects of HBV infection. The study contributes in narrowing the existing gap of HBV molecular study in Ethiopia.

## Introduction

Hepatitis B virus (HBV) infection remains one of the major worldwide public health burdens. Global estimates suggest that more than 2 billion people have been infected with HBV, and250 million of these people are chronically infected, of which 65 million live in Africa[1–3]. HBV prevalence is highest in Sub-Saharan Africa and East Asia, where between 5–10% of the adult population is chronically infected. It accounts for 500,000–1.2 million deaths per year and is the tenth leading cause of mortality worldwide[4, 5]. Prevalence of HBV in Ethiopian blood donors showed marked variations in different parts of the country, with a pooled prevalence of 8.4% [6].

HBV belongs to the *Hepadnaviridae* family and contain a partially double-stranded circular DNA genome, approximately 3.2kb length. It presents four partially overlapping open reading frames encoding: polymerase, surface, core and X proteins [7–9]. HBV replicates through a reverse-transcribed RNA intermediate by reverse transcriptase enzyme, which has no proof-reading capabilities that leads to highly error-prone nucleotide synthesis during viral replication [10, 11]. HBV exhibits genetic variability with an estimated rate of 1.4–3.2 x 10^−5^ nucleotide substitution per site per year [12]. The genetic variability has resulted in emergence of ten HBV genotypes (A-J) differ in more than 8% of the genome [13, 14], while forty sub-genotypes differ at least by 4% of the genome [10, 14].

HBV genotypes play a role in both course of infection and treatment management [15]. Genotype based structural and functional differences can influence the severity, course and likelihood of complications, and response to treatment of HBV infection and possibly vaccination against the virus[13, 16]. In addition to HBV genotype diversity, genomic variation on Hepatitis B Surface Antigen (HBsAg) has led to description of mutations with considerable effect. Mutations within the HBsAg central region namely Major Hydrophilic Region (MHR, a.a. 99–169); have been shown to be related with failure of HBsAg detection, antiviral resistance and vaccine escape [17, 18]

Genotypes and sub-genotypes of HBV are distributed worldwide although some are localized to particular geographic regions [10, 19]. Sub-Saharan Africa is one of the highly endemic regions for HBV; with genotypes A, D, and E found in diverse geographical locale. However, there is limited information on prevalent genotypes in many African countries [3]. Genotype A is found in Southern-Eastern Africa [20–22]. Genotype D is found throughout Africa but appears to prevail predominantly in northern African countries [23, 24]. Genotype E is found mainly in countries of Western Africa [25, 26].

Ethiopia is a country with high hepatitis B carrier prevalence and the burden of HBV likely remains significant; however, its genotype pattern, genetic diversity and mutation analysis is largely unknown. The Ethiopian Ministry of Health issued national strategy for prevention and control of viral hepatitis and, since 2016, antiviral therapy has been available to patients with chronic hepatitis B at selected hospitals and clinics in the country[27–29]. To the best of our knowledge, there was no HBV genotype study in Ethiopia until the end of 2015. Recently, an Ethiopian study[30] showed an overall distribution of 78% genotype A and 22% genotype D, while another study [31] from Northern Ethiopia reported genotype A (58.4%), genotype D (41%) and genotype E (0.6%); but neither of both studies assessed the full HBV serological markers and biochemical profiles in relation with HBV genotype. Moreover, the distribution of genotypes may vary between geographical regions within a nation as observed in other East African neighboring countries like Sudan [32, 33] and Kenya [22, 34]. Therefore, the present work was conducted to determine HBV genotypes distribution alongside mutation profiles and sero-virological characteristics among hepatitis B infected blood donors in Southern part of Ethiopia.

## Methods

### Study design and samples

This was a cross-sectional, laboratory based study with convenience sampling technique. A total of 2,237 blood donors were screened for HBV, HCV, HIV and syphilis (*Treponema pallidum*)as part of routine blood donation requirement at Hawassa Blood Bank, Southern Ethiopia; between May and December 2016. Blood donors who fulfilled the national blood bank criteria to be eligible for donation were included. One hundred three donors mono-infected with HBV were used for the present study and serum samples obtained from these donors coded with unique identifier number and stored at -56°C until use.

### Ethical clearance

The Institutional Review Board of College of Medicine and Health Sciences (Ethiopia)and Biomedical Ethics Committee of Anhui Medical University (China) initially permitted the study protocol. Final approval and ethical clearance of the study was obtained from National Research Ethics Review Committee (NRERC), Ministry of Science and Technology, Ethiopia. A written informed consent was obtained from all participants.

### Serological assays

Screening of HBsAg, anti-HCV and anti-*T*.*pallidum* was done using an Enzyme Linked Immuno-sorbent Assay (ELISA) kits (DIALB Diagnostics GmbH, Vienna, Austria) and for HIV screening Vironostika HIV Uni-Form II Ag/Ab ELISA kit (Bio-Merieux, Boxtel, Netherlands) was used. HBsAg-positive serum samples from donors co-infected either with HIV or HCV were excluded from the present study.

Serum samples were re-tested for HBsAg, and screened for other HBV serological markers (Hepatitis B envelop antigen [HBeAg], Hepatitis B envelop antibody [HBeAb], Hepatitis B core antibody [HBcAb], Hepatitis B surface antibody [HBsAb]) using ELISA kits (Zhongshan Bio-Tec., Guangdong, China) as instructed by manufacturer in Addcare ELISA 1100 analyzer (Yantai Addcare, Shandong, China).

### Biochemical assays

Levels of serum alanine transaminase (ALT) and aspartate transaminase (AST) were measured using a commercial kit (Roche Diagnostics, Switzerland) in automated clinical chemistry analyzer. The upper limit of normal (ULN) values for ALT and AST was 40 IU/L and 35IU/L, respectively.

### DNA extraction

Viral DNA was extracted from 200μL of serum using the QIAamp DNA Blood Mini Kit (QIAGEN GmbH, Hilden, Germany) according to the manufacturer’s instructions, and eluted in 100μl buffer. Eluted DNA samples were stored at -20°C until use.

### Viral load quantitation

Hepatitis B viral load quantified by SLAN 96S Real-Time PCR System (Hongshi Med.Tec., Shanghai, China) using Liferiver HBV Quantitative Real Time PCR kit (ZJ Bio-Tec, Shanghai, China) with lower detection limit of 20 IU/ml. Internal Quality Control samples supplied with kit were utilized.

### Amplification of HBV S-gene

Nested PCR was performed to amplify partial HBV S-gene (nucleotide 231–801) as described previously[35] with slight modifications. This part of the S-gene we chose has MHR and is highly conserved sites, so it could be used in HBV genotyping, serotyping and analysis of mutation. The first PCR was done using primers PS1F (5’-TCACAATACCGCAGAGTCT-3’) and PS1R (5’-AACAGCGGTATAAAGGGACT-3’). Second PCR was carried out with primers PS2F (5’-GTGGTGGACTTCTCTCAATTTTC-3’) and PS2R (5’-CGGTATAAAGGGACTCACGAT-3’) to obtain an amplified product of 541bp. All primers were obtained from Sangon Biotech Company (Shanghai, China) and TaKaRa Pre-mix Taq (Shanghai, China) was used for PCR. Amplification conditions were initial denaturation at 94°C for 3min, followed by 40 cycles of 30sec at 94°C denaturation, 30sec at 55°C annealing, and 1min at 72°C extension, followed by a final extension of 5min at 72°C; using T100 Thermal Cycler (Bio-Rad Lab, Singapore). Cycling parameters for the second PCR remained the same as in the first one except that the number of cycle was reduced to thirty. In order to prevent PCR carryover contamination strict care and procedures implemented as previously mentioned [36].

### DNA sequencing

The BigDye Terminator v3.1 Cycle Sequencing Ready Reaction Kit was used for bi-directional sequencing with an ABI PRISM 3730 DNA Analyzer (Applied Biosystems, CA, USA). The second pair of primer mentioned above (PS2F and PS2R) was used for sequencing.

### Sequence analysis and genotyping

Nucleotide BLAST was performed on our HBV sequences to acquire a homologous sequence from NCBI. Multiple sequence alignment was conducted using ClustalW implemented in MEGA-7[37]. HBV genotype was determined with phylogenetic analysis of aligned sequences in comparison to HBV reference sequences retrieved from NCBI. Phylogenetic tree was constructed based on Neighbor-Joining method with Kimura-2 parameter model in conjunction with estimation of the tree reliability by bootstrap method of 1000 replicates; all of these processes were done using MEGA-7[37]. Visualization of the tree was done by *FigTree* v.1.4.3 software. For independent cross-checking, a web-based NCBI genotyping tool [38]was used. Recombinant analysis was performed by online recombination detection method of jpHMM[39]. The accession numbers of HBV isolates sequenced in this study have been deposited in GenBank asMF169791 to MF169875.

### HBV serotyping

Aligned DNA sequences were translated to corresponding amino acid sequences using MEGA-7[37], and became ready for serotyping. HBV serotypes were assigned based on the amino acids present at either three or five known specific position (a.a. 122,160,127,159,140) on HBsAg as described previously [40].

### Mutation analysis

Amino-acid sequences obtained from translation of DNA sequences were compared with collection of reference amino-acid sequences of similar genotype retrieved from GenBank, to analyze presence of mutation within and outside the MHR. The mutation pattern was assessed based on published reports [41, 42]using BioEdit *ver*.7.2.5; for further confirmation, an online tool *Geno2Pheno* was also used.

### Statistical analysis

SPSS *ver*. 20 (IBM Corporation, Armonk, NY, USA) was used as tool for statistical analysis. Categorical data were analyzed using the Chi-square test or Fisher’s exact test if appropriate. Mann-Whitney U test was used to compare continuous variables. A two-tailed *p*-value < 0.05 considered statistically significant.

## Results

A total of 2,237 blood donors were screened, the sero-positivity for HBV, HIV, HCV, and syphilis were 106(4.7%), 38(1.7%), 11(0.5%), and 11(0.5%), respectively. Of 106 HBsAg sero-positive donors three had co-infection (2HBV-HIV and 1HBV-HCV); these three donors with co-infection were excluded from the study. One hundred three serum samples collected from HBV mono-infected blood donors were used for further laboratory analyses. Majority of the donors (84.5%) were males. The median age of study participants was 25years, with a range of 18 to 55 years. The donors consisted mainly of voluntary donor type and were not receiving HBV antiviral therapy as they responded during the pre-donation interview. In Table 1 baseline characteristics of study participants summarized.

**Table 1:** Demographic and laboratory profiles of HBsAg sero-positive blood donors.

Characteristics	Values
**Gender** *(Male*: *Female)*	87:16
**Age** *(Years)*	25(21–30)
**HBeAg positive**,*n (%)*	15(14.6)
**HBeAb positive**,*n (%)*	73(70.9)
**ALT** *(IU/L)*	21(17–29)
**AST** *(IU/L)*	30(25–40)
**Viral Load** *(log IU/ml)*	3.46(2.62–4.82)
**HBV Serotype**	
**- adw2**,*n(%)*	63(74.1)
**- ayw2**,*n(%)*	21(24.7)
**- ayw3**,*n(%)*	1(1.2)
**HBV Genotype**	
**- HBV/A**, *n (%)*	58(68.2)
**- HBV/D**, *n(%)*	27(31.8)

All continuous quantitative data are expressed as Median (Inter-quartile Range), M (IQR)

The median (interquartile range/IQR) serum levels of ALT and AST was [21 (17–29)] IU/L and [30 (25–40)] IU/L, respectively. Five (4.9%)donors had ALT value above 1.5xULN, while nine (8.7%)had AST level more than 1.5xULN. Serum AST level was significantly higher in HBeAg positive samples than in HBeAg negative samples (median: 41IU/L *vs* 29 IU/L, respectively) (*p* = 0.001). No association was found in the serum ALT levels with regard to demographic and HBV sero-markers.

### HBV serology and viral load

All sera were HBsAg and HBcAb reactive, while HBsAb non-reactive. HBeAg and HBeAb presence was tested in all of the samples. HBeAg was detected in 15 (14.6%) samples, and 73 (70.9%) samples were reactive for HBeAb. None of the samples had simultaneous presence of HBeAg and HBeAb, whereas 15 sera had neither HBeAg nor HBeAb.

HBV DNA load was quantified in 98/103 (95.1%) samples, and the median (IQR) was 3.46(2.62–4.82) log IU/ml. In five (4.9%) sera, undetectable viral load result was obtained. Forty-three (43.9%) samples had viral load below 3.3log IU/ml, while 30 (30.6%) of samples had viral load above 4.3 log IU/ml. The median viral load of HBeAg-positive samples [6.89(6.26–7.32)log IU/ml] was significantly (*p*<0.001) higher than in HBeAg-negative samples [3.20 (2.50–4.13) log IU/ml].

### HBV genotype

**Fig 1:**
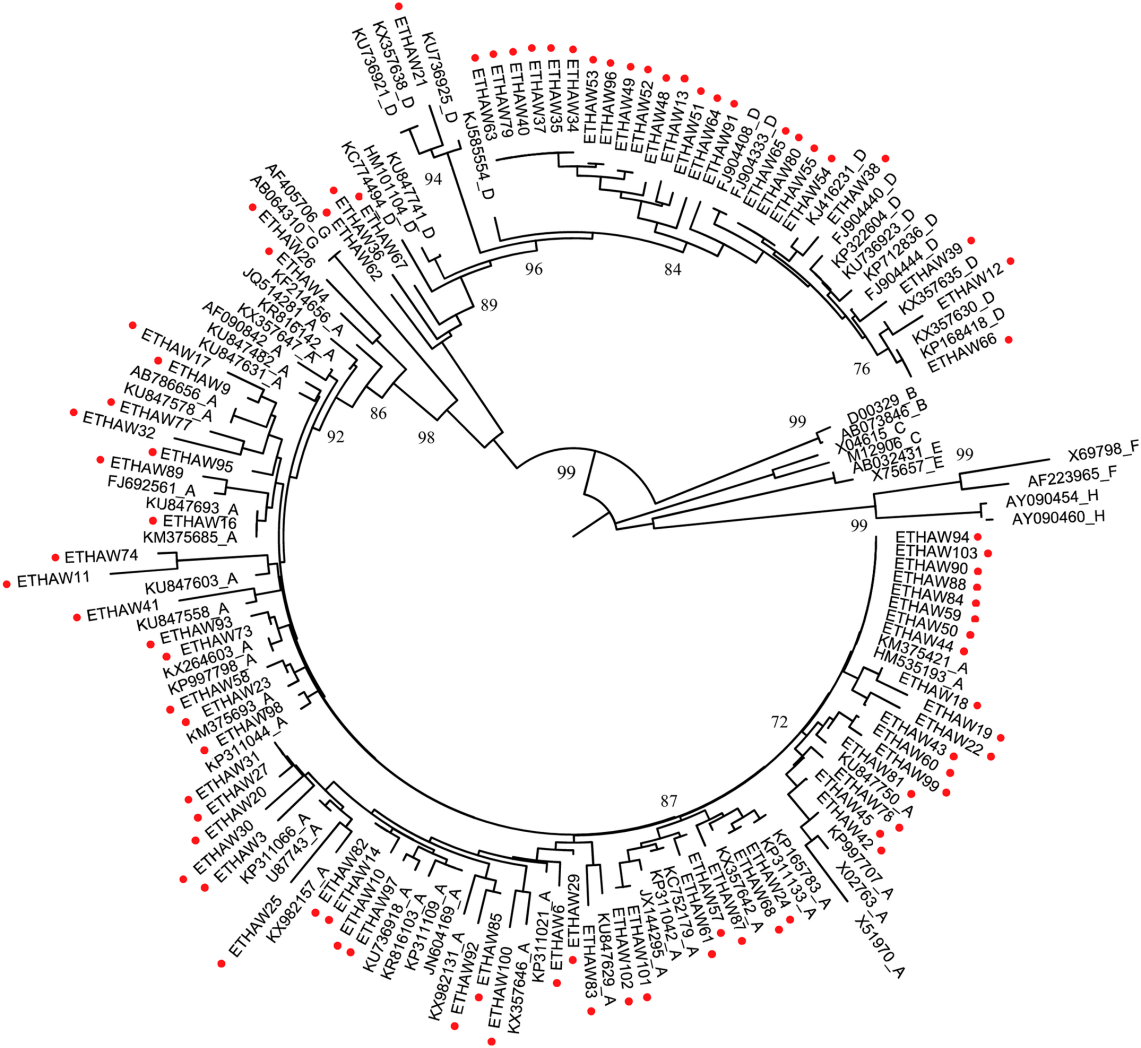
Neighbor-Joining rooted phylogenetic tree of partial HBV S-gene (231–801 nt) sequences from 85 Ethiopian isolates start with ETHAW and marked by red-dot (●) and 71 reference sequences retrieved from GenBank with accession number followed by respective HBV genotype A to H. The numbers at the nodes indicate percentage of bootstrapping values.

HBV partial S-gene was successfully amplified and sequenced from 85 of 98 samples with detectable viral load result. Based on phylogenetic analysis of our 85 isolate sequences with reference sequences obtained from NCBI (Fig 1); 58 (68.2%) of the isolates classified into genotype A (HBV/A), while 27 (31.8%) of isolates categorized into genotype D (HBV/D). All of the sequence isolates from this study undergone recombination analysis, but none of them had recombinant strain.

Twenty seven (27/58; 45.5%) of genotype A donors had a VL above4.3 log IU/ml, while only three (3/27; 11.1%) of genotype D donors had VL more than 4.3 log IU/ml; and the variation observed was significant (*p* = 0.002). The rate of HBeAg reactivity in genotype A subjects appeared higher in comparison with genotype D donors (13/58 *v*s 2/27), but the difference was not statistically significant. No significant differences were seen between genotypes with regard to donor’s age, gender or biochemical assay results (Table 2).

**Table 2:** Comparison of HBV genotype A and genotype D infected donors with demographic and laboratory profiles.

Characteristics	Genotype A (HBV/A)(n = 58)	Genotype D (HBV/D)(n = 27)	*p-value*
**Gender** *(Male*: *Female)*	48:10	23:4	0.999
**Age** *(Years)*	26(20–30)	23(21–30)	0.667
**HBeAg positive**,*n (%)*	13(22.4)	2(7.4)	0.129
**HBeAb positive**, *n (%)*	36(62)	22(81)	0.073
**ALT** *(IU/L)*	21.5(16.7–33.0)	22(18–24)	0.769
**AST** *(IU/L)*	34(26.7–46.5)	30(26–35)	0.093
**Viral Load** *(log IU/ml)*	4.15(3.26–6.26)	3.11(2.51–3.98)	0.005*
**HBV Serotype**			
** - adw2**, *n (%)*	58(100)	5 (18.5)	<0.001*
** - ayw2**, *n (%)*	0	21(77.8)	
** - ayw3**, *n (%)*	0	1(3.7)	
**MHR Mutation**, *n (%)*	21 (36.2)	3(11.1)	0.013*

All continuous quantitative data are expressed as Median (Inter-quartile Range), M (IQR)

*denotes statistically significant *p-value*(< 0.05)

### HBV serotype

The serotype distribution of the 85 isolates based on their deduced amino-acid sequences was 63 (74.1%)*adw2*, 21 (24.7%) *ayw2*, and 1(1.2%) *ayw3*. All of genotype A samples were categorized into *adw2* serotype, while genotype D samples were classified to *ayw2*(21/27), *adw2*(5/27) and *ayw3*(1/27) serotypes. Distribution of *adw2* serotype was significantly higher among HBV/A donors than HBV/D donors (*p*<0.001). HBV serotypes distribution was compared with respect to donor’s age, gender and biochemical assay; the difference was statistically insignificant.

### Mutation in HBV S-region

**Fig 2:**
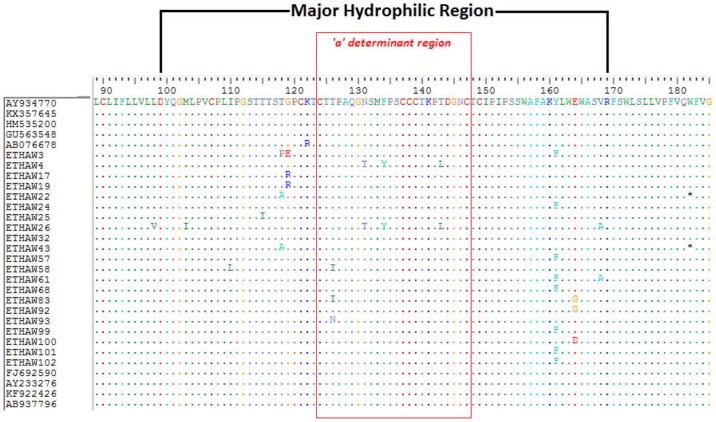
Distribution of amino acid substitutions (mutations) detected within the major hydrophobic region (MHR) of HBV S-gene among 21 genotype A isolates aligned with nine reference sequences retrieved from GenBank.

In a total of 24(28.2%) isolates either one or more mutation was detected within the MHR. Six (7.1%)of these isolates had amino-acid substitutions (T126I/N, N131T, M133V, F134Y and T143L) within the ‘a’ determinant region (a.a124-147) of the MHR (Table 3 and Fig 2). The occurrence mutation within MHR was significantly higher in genotype A than genotype D (36.2% *vs* 11.1%; *p* = 0.013). The median (IQR) VL and ALT level of donors with MHR mutation was 3.75 (2.91–6.23) log IU/ml and 20 (14–33) IU/L, respectively.

**Table 3:** Distribution of mutation in partial HBV S-gene among isolates from blood donors.

HBV S-gene Region		Mutation	Sample/Sequence Identification
MHR (a.a 99–169)	‘a’ determinant region(a.a. 124–147)	T126I/N	ETHAW58, ETHAW83, ETHAW93
		N131T	ETHAW4, ETHAW26
		M133V	ETHAW13
		F134Y	ETHAW4, ETHAW26
		T143L	ETHAW4, ETHAW26
	Outside ‘a’ determinant region(a.a. 99–123 & 148–169)	M103I	ETHAW26
		I110L	ETHAW32, ETHAW58
		T115I	ETHAW25
		T118A /P	ETHAW-3, 21, 22, 43
		G119 E/R	ETHAW3,17, 19
		Y161F	ETHAW-3, 24, 68, 57, 61, 99, 101, 102
		E164D/G	ETHAW-83, 92, 100
		V168A	ETHAW-26, 34
Downstream of the MHR (a.a.170-200)		V177A	ETHAW87
		W182*	ETHAW22, ETHAW43
		V184A	ETHAW77, ETHAW95
		T189I	ETHAW-21, 25, 85, 92

*denotes stop codon

Further analysis of the amino-acid sequences in the downstream of the MHR (a.a 170–200) was performed for detection of other mutations. In 7 (4.7%) of samples a known amino acid substitutions (V177A, V184A, T189I) were observed (Table 3). In two isolates (ETHAW22 and ETHAW43) a stop codon (W182*) was detected; both of these two isolates were from HBV/A and HBeAg-negative with viral load of2.15log IU/ml and 5.49 log IU/ml, respectively.

## Discussion

Africa is one of the highly endemic regions of HBV and three genotypes (A, D, and E) have been identified in different countries[3]. HBV genotypic and serologic patterns might help in the designing of management plans, predicting clinical outcomes and updating prevention strategies[43]. Ethiopia is a country with high hepatitis B carrier prevalence and neighbored by countries with variety of HBV genotypes distribution. However, very limited report regarding the molecular study of HBV in Ethiopia was available. Therefore, in this study we determined genotype distribution, mutational pattern and sero-virological profiles of HBV isolates from blood donors in Southern Ethiopia.

Our study demonstrated the presence of genotype A (68.2%) and genotype D (31.8%) in blood donors. The predominance of genotype A is consistent with recent HBV genotype distribution studies in Ethiopia [30, 31]. The result of genotype A almost found in the middle of these two Ethiopian studies reported 78% and 58.4%, respectively. Genotype D distribution of our study is also comparable to certain extent with aforementioned two studies. The HBV genotypes show a distinct geographical distribution in Africa, with genotype A predominating in the South-East, genotype D in the North and genotype E in the West [13]. HBV genotype A has been reported to be predominant in Kenya [22, 34], Uganda [44] and Tanzania [21], while genotype D is prevalent in Sudan [32, 33] and Egypt [23]. It can be inferred that Ethiopia is found at the presumed geographical junction to distribution of these two HBV genotypes. Even though genotype E was reported from Ethiopia[31]and neighboring countries [22, 32], in our case no genotype E was identified.

In addition to genotyping, HBV isolates can be categorized into serological subtypes (serotypes) based on amino acids present at specific positions[13]. In our study the most prevalent HBV serotype identified was *adw2* (74.1%) followed by *ayw2* (24.7%) and *ayw3* (1.2%). All of genotype A samples serotyped as*adw2*; while genotype D samples were serotyped as *ayw2*, *adw2* and *ayw3*. Our finding is in agreement with association between HBV serotype and genotype as stated previously [45]. The current result is also consistent with other study[30]by reporting the predominance of *adw2* in Ethiopia and serotyping of all genotype A samples into *adw2*; but difference was seen on the frequency and distribution of *ayw2* and *ayw3*.

Knowing about the predominant HBV molecular variants present at specific area is significant for the assessment of diagnostic capabilities and vaccine efficacy [46]. Mutations at MHR have been shown to be related with diagnostic problems, emergence of vaccine-escape mutants, and hepatitis B immunoglobulin (HBIG) therapy failure[17, 18, 42]. The mutation analysis of our study showed that 24(28.2%) donors had amino-acid substitutions within the MHR, six of which occurred at ‘a’ determinant site. Our finding is slightly higher than[30, 47], and lower than [34] studies conducted on HBV infected blood donors. Another study conducted on HBV/HIV mono or co-infected Ethiopians reported occurrence of immune-escape mutants at different level[31]. Amino-acid substitutions at MHR are associated with immunological pressure resulted from both natural and HBV vaccination [17, 42]. In Ethiopia, the hepatitis B vaccine has been introduced in 2007[29]. In our study vaccination history of donors was not available, but donors in this study were all born earlier than the year of national vaccination launched. However, the finding of immune escape variants in ours and prior Ethiopian molecular studies[30, 31]should be taken into account as there is a possibility for these variants to spread more and consequently influence vaccine efficacy and treatment strategy in the country.

The presence of HBV genotype A and D in our study subjects provided the access to compare different parameters in stratified way with respective genotype (Table 2). Donors infected with genotype A carried higher (4.15 log IU/ml *vs* 3.11 log IU/ml) viral load than those infected with genotype D as observed in Polish study [47] but on contrary to other report[16]. When assessing HBeAg seropositivity among these two genotypes, there were more HBeAg-positive samples in genotype A subjects compared with genotype D, although the difference was not statistically significant. One review[16] stated that HBV/A was more prevalent in HBeAg-positive chronic hepatitis patients, whereas genotype D was more prevalent in those positive for HBeAb. In the current study, further comparison showed that donors infected with HBV/A had significantly more frequent MHR mutation than HBV/D infected donors, as also reported in Kenya [34] but incongruent with other study [47]. As observed in earlier reports [43, 48]age, gender and liver enzymes levels were not significantly different among genotypes.

In the present work, fifteen (14.5%) donors were positive for HBeAg and comparable with other studies in Tanzania [21], Egypt [49], and Poland[47] but lower than reports from Ethiopia[50]and Benin [51]. Seropositivity for HBeAg, a marker of active viral replication and high infectiousness, is associated with higher risk for hepatocellular carcinoma; and it is significant regardless of serum level of ALT and status of liver cirrhosis[52]. In the course of chronic hepatitis B (CHB) infection, sero-conversion to HBeAb has been associated with changes in HBV DNA replication rate and clinical status. HBeAg is often associated with active and progressing liver disease, whereas sero-conversion to HBeAb frequently concurred with considerable decline of viral DNA[53]. Our finding also showedthatviral load of HBeAg positive/HBeAb negative samples significantly (*p*<0.001) higher than HBeAg negative/HBeAb positive samples, as observed in prior studies from blood donors [21, 54].

In our study, ninety-eight (95.1%) of donors had ALT level below 1.5xULN, it is comparable to data reported in asymptomatic HBV carrier blood donors [15, 47, 49]. Those donors who had elevation of ALT level above 1.5xULN showed also higher viral load value, even if the relation was statistically insignificant (*p* = 0.060). In individuals with CHB the role of ALT levels as a predictor of liver injury has been questioned. HBV infected persons with persistently normal ALT but active viral replication may have clinically significant liver disease [55].

It is known that all our isolates were obtained from ‘apparently healthy’ individuals in blood donor settings and screening of HBV was done for safe blood transfusion. Generally donors identified as reactive for HBV infection during blood screening are not monitored and there is no management of the infection. Hence in future better to explore the clinical settings with patients if the situation is different and to further elucidate the impact of diversity in HBV infections and disease management.

In summary, the current study illustrated distribution of HBV genotypes with mutation profiles and sero-virological characteristics among blood donors in southern Ethiopia. The predominant genotype and serotype were HBV/A and *adw2*, respectively. HBeAg sero-positive donors carried more viral load than sero-negative donors. Donors infected with genotype A had higher viral load and more frequent MHR mutation than those infected with genotype D. We have also demonstrated the occurrence of HBV variants that may influence clinical aspects of its infection. Therefore, our study will become an input in narrowing the existing gap of HBV molecular study in Ethiopia.
